# Serious Games Integrating Perceptual Learning and Stereopsis Training in Children With Amblyopia: Single-Arm Pre-Post Feasibility Study

**DOI:** 10.2196/77402

**Published:** 2025-12-17

**Authors:** Yiwei Mo, Peng Chen, Manting Hou, Fang Cui, Chao Luo, Chao Deng, Jinkang Lin, Qi Wu

**Affiliations:** 1 School of Digital Media, Shenzhen Polytechnic University, Room 321, Block A, Zhixingyuan, Liuxiandong Campus, 7098 Liuxian Avenue, Nanshan DistrictShenzhen, Guangdong, 518111, China, +86-188-2026-1619; 2 School of Visual Arts Design, Hubei Institute of Fine Arts, Wuhan, Hubei, China; 3 School of Psychology, Shenzhen University, Shenzhen, Guangdong, China; 4 School of Art and Humanities, Hubei Institute of Fine Arts, Wuhan, Hubei, China

**Keywords:** amblyopia, serious games, perceptual learning, stereopsis training, visual function improvement

## Abstract

**Background:**

Amblyopia, a leading cause of preventable childhood blindness, often remains inadequately addressed by traditional treatment methods such as refractive correction and occlusion therapy, which can be noninteractive and lead to poor adherence.

**Objective:**

This study aimed to design and evaluate the feasibility and preliminary efficacy of a serious game intervention that integrates perceptual learning and stereoscopic vision training for pediatric amblyopia and to explore its potential as a complementary or alternative approach to conventional treatments such as occlusion or atropine therapy.

**Methods:**

We evaluated visual acuity, accommodative sensitivity, binocular accommodation, stereopsis, and compliance-related data in a cohort of children with amblyopia aged 7 to 12 years before and after a 3-month intervention. Participants engaged in visual training via a serious game, attending sessions 4 times weekly for 30 minutes each.

**Results:**

Best-corrected visual acuity improved significantly from 0.42 (SD 0.16) to 0.37 (SD 0.18) logMAR, yielding a mean difference of 0.05 (95% CI 0.03‐0.08; *t*_24_=4.82; Cohen *d*=0.96; *P*<.001). Accommodative sensitivity showed marked enhancement following the intervention. In the amblyopic eye, mean values increased from 1.44 (SD 2.18) to 4.96 (SD 2.91) cycles per minute, corresponding to a mean change of −3.52 (95% CI −4.59 to −2.45; *t*_24_=−6.81; Cohen *d*=−1.36; *P*<.001). Under binocular viewing, accommodative sensitivity improved from 1.52 (SD 2.49) to 5.08 (SD 2.71) cycles per minute (Δ=−3.56, representing the mean paired difference (baseline minus post), 95% CI −4.88 to −2.24; *t*_24_=−5.56; Cohen *d*=−1.11; *P*<.001). Stereoacuity also improved significantly, decreasing from 780.0 (SD 613.6) to 448.8 (SD 472.2) arc sec (Δ=331.20, 95% CI 134.20‐528.20; *t*_24_=3.47; Cohen *d*=0.69; *P*=.002). The proportion of participants with normal Worth 4 dot responses increased from 76% (19/25) at baseline to 96% (24/25) after the intervention, and treatment adherence was high under supervised clinical conditions.

**Conclusions:**

The integration of gamified elements into amblyopia treatment was associated with high adherence under supervised clinical conditions and with significant short-term improvements in visual function, suggesting a promising complementary approach to conventional therapies. This interactive approach effectively combines perceptual learning with stereopsis training, presenting a potential alternative to conventional therapies.

## Introduction

Amblyopia is a cortical visual impairment clinically manifested as a unilateral or bilateral reduction in visual acuity (VA) that cannot be explained by structural abnormalities of the eye [1]. It has an estimated prevalence of approximately 3.4% in children [2] and poses a significant risk of lifelong visual loss [3]. The first-line treatment is refractive correction [4]; if this proves ineffective, occlusion therapy is commonly implemented [5]. Although occlusion is considered the standard treatment, it lacks binocular visual stimulation and is often associated with poor adherence [6,7]. Atropine may serve as an alternative intervention, but it may cause systemic side effects [8].

Advancements in our understanding of neurophysiological mechanisms have led to perceptual learning (PL) based on visual stimuli garnering increasing attention and emerging as a therapeutic approach for amblyopia treatment [9,10]. PL refers to the neuroplastic process induced by specific training that leads to persistent functional optimization of perceptual systems. It relies on the neuroplasticity of the visual cortex [11], enabling the visual system to dynamically adapt to task demands and trigger neuroplastic changes that enhance visual performance [11,12]. Clinical studies have confirmed that this training paradigm can significantly improve multiple dimensions of visual function in patients with amblyopia, including positional acuity [11,13], stereopsis [13], contrast sensitivity, and VA [14], while inducing long-term functional modifications [15].

Existing neuroimaging studies suggest that PL may be accompanied by plastic changes within the visual cortex and associated networks, such as alterations in the activity of primary and secondary visual areas and remodeling of white matter tracts. Moreover, certain gamified training paradigms have been linked to structural and functional modifications in additional brain regions involved in spatial navigation, executive control, and motor coordination. It should be emphasized that this study did not include neuroimaging assessments, nor did it directly measure these neural mechanisms. Therefore, the mechanistic statements below are presented solely as general interpretations based on previous literature, intended to provide a theoretical context for the design of the training tasks, rather than as causal explanations of the findings in this study.

Neuroimaging studies have provided direct evidence for the neuroplastic mechanisms underlying PL. Using multimodal imaging techniques, Zhai et al [16] found that 30 days of PL intervention induced significant enhancement of neural activity in the primary visual cortex (Brodmann areas 17‐19) and bilateral temporal lobes of patients with amblyopia, accompanied by structural remodeling of white matter tracts. Notably, training paradigms incorporating gamified elements have been shown to selectively increase gray matter volume in the right hippocampus (associated with spatial navigation), prefrontal cortex (executive control center), and cerebellum (motor coordination hub) [17]. These findings not only confirm the persistent neuroplasticity of the adult brain but also provide neuroanatomical validation for the theory that cognitive training enhances higher-order brain functions. Current advancements highlight the dual value of PL in visual system remodeling [18,19] and amblyopia therapeutics. Clinical evidence demonstrates that synergistic integration of PL with traditional occlusion therapy significantly accelerates visual rehabilitation in adolescent amblyopes [20].

Stereopsis, as the ability of the 2 eyes to work together to perceive depth and spatial position [21], is one of the important visual functions in amblyopia treatment. Its formation relies on the brain’s processing of parallax—the image offset received by the left and right eyes due to spatial position differences. The parallax effect is the core physical basis of stereoscopic vision, and by controlling this parallax amount, the brain is able to merge the 2D images and reconstruct 3D spatial information [22,23]. In amblyopia, interocular suppression disrupts binocular fusion mechanisms, leading to stereoscopic deficits [24]. Clinically, children with amblyopia with impaired stereopsis face a 2.2-fold higher risk of persistent amblyopia compared with those with intact binocularity [25].

Recent years have seen increasing interest in binocular and gamified interventions. In adults, Vedamurthy et al [26] showed that a dichoptic action video game significantly improved VA, contrast sensitivity, and stereopsis, with retention after a no-contact period. In children, however, the results are more cautious. The Pediatric Eye Disease Investigator Group indicated in a multicenter randomized controlled trial (RCT) that the Falling Blocks binocular iPad game (provided by the research team) did not outperform spectacle correction alone after 8 weeks, largely due to poor adherence [27]. Nevertheless, promising findings continue to emerge. Bari et al [28] demonstrated in Italian patients that a home-based binocular rehabilitation using red-blue anaglyphic games significantly improved best-corrected visual acuity (BCVA) within 8 weeks. Bocqué et al [29] found that the updated FAVAS 2020 platform significantly increased adherence compared with its 2015 version. In China, Zhu et al conducted a prospective pilot study in preschool children, showing that gamified binocular therapy significantly improved both distance VA and stereoacuity after 12 weeks, with good safety [30].

Virtual reality (VR) technologies are also being tested. Hirota et al [31] evaluated a motion-based VR dichoptic training system in healthy adults and confirmed its safety, with minimal ocular or systemic side effects. Moreover, Li et al [32] showed that stereoscopic 3D video games enhance the precision, but not the accuracy, of depth perception, highlighting the mechanism through which disparity processing can be optimized. Together, these studies suggest that amblyopia management is shifting from “occlusion and penalization” toward “PL, gamification, and VR.”

Finally, amblyopia prevention also requires innovations in screening and triage pathways. Morse and Oatts [33] emphasized that current screening technologies are inconsistent, and new strategies—such as artificial intelligence–assisted diagnostics and biomarker-based approaches—are urgently needed to optimize early detection and referral. This systemic perspective provides a rationale for integrating low-cost anaglyph glasses with touchscreen devices in community and home-based training settings.

Within this evolving landscape, particular attention has turned to children in late childhood. Although ages 7‐12 years are traditionally viewed as approaching the tail end of the sensitive period for amblyopia treatment, converging evidence indicates that residual visual cortical plasticity can be harnessed beyond early childhood [34,35], especially when interventions incorporate binocular integration, contrast rebalancing, and PL-based tasks. Studies in older children, adolescents, and adults have reported meaningful, though sometimes attenuated, gains with binocular, game-based, or PL interventions [34,36]. At the same time, clinical trials in 7‐12-year-olds indicate that conventional patching or atropine can still yield improvements, but with lower overall success rates, residual deficits, and adherence challenges compared with younger children [37,38]. Taken together, these findings suggest that 7‐12-year-olds constitute a clinically relevant target group in which engaging, technology-supported binocular training warrants systematic evaluation rather than being dismissed as beyond therapeutic reach.

Based on these advances, this study introduces a gamified serious-game framework integrating PL and stereopsis training, using red-blue anaglyph glasses and tablet devices to generate controlled binocular stimuli. The aim is to explore the therapeutic potential of this approach in children aged 7‐12 years with amblyopia, focusing on improvements in VA, accommodative function, and stereopsis, while positioning the intervention as a potential complement to conventional therapies. Given these gaps in the current evidence, several key research questions remain.

First, can a gamified intervention that integrates PL principles with stereopsis training demonstrably improve multiple dimensions of visual function in children with amblyopia—including VA, accommodative sensitivity, binocular accommodative function, and stereopsis—within a clinically feasible time frame?

Second, without direct comparison to conventional therapies (such as occlusion or atropine), does this intervention demonstrate feasibility as a potential supplement or alternative to standard treatment (eg, improving compliance and potentially providing a more accessible, lower-cost digital pathway)?

Third, how might objective adherence data captured through digital platforms be leveraged to refine individualized treatment protocols and to inform the design of future RCTs?

By addressing these questions, this study aims to provide preliminary clinical evidence for the feasibility, safety, and efficacy of gamified binocular digital therapy and to lay a foundation for subsequent large-scale, multicenter investigations.

## Methods

### Ethical Considerations

This study was a single-group, pre-post feasibility design. A “no-treatment” or “delayed-treatment” control group was not included because, during ethical review and discussions with parents, a substantial proportion of guardians declined to have their children randomly assigned to a waiting or nonintervention group. In accordance with routine clinical practice and the recommendations of the ethics committee, establishing a waiting group or suspending intervention was considered inappropriate under the current condition of insufficient clinical equipoise. To balance acceptable risk and practical feasibility, the study adopted a single-group intervention approach to evaluate feasibility and identify preliminary efficacy signals, with the following prespecified commitments and risk control measures.

First, refractive correction was maintained and standardized throughout the study (standard care was not interrupted).

Second, rescue criteria were established; if adverse events, new-onset diplopia, or significant changes in ocular alignment, or substantial deterioration in visual function relative to baseline occurred (thresholds predefined in the protocol), the training was immediately discontinued, and conventional treatment was resumed.

Third, during the intervention, occlusion or atropine therapy was not initiated proactively. If clinically required, initiation or adjustment was made independently by the attending ophthalmologist and recorded as a protocol deviation, which would be included in sensitivity analyses.

Finally, statistical analyses were limited to within-group changes and correlations without making any claims of superiority or inferiority relative to occlusion or atropine treatment.

The research protocol adhered to the principles of the Declaration of Helsinki and was approved by the Human Research Ethics Committee of the Faculty of Psychology, Shenzhen University (SZU_PSY_2024_100). After being fully informed of the study objectives, procedures, potential risks, and alternative treatment options, written informed consent was obtained from the guardians, while children provided assent or consent appropriate to their age and level of understanding. Participants were free to withdraw from the study at any time without any impact on their clinical care. No monetary or material incentives were offered. Data collection followed the principle of minimum necessity, using deidentified coding with restricted access. No sensitive personal information was collected, and all data were used solely for research purposes.

### Participants

Participant recruitment began in July 2024, with intervention initiated in September 2024 and continuing for 12 weeks. All training sessions and data collection were completed by December 2024. A total of 25 children (Table 1) were enrolled in the study, including 9 males, all recruited from Luohu People’s Hospital, Shenzhen. The mean age was 8.04 (SD 1.457) years. Among the participants, 22 had anisometropic amblyopia, and 3 had strabismic amblyopia. Based on treatment history, 12 children had never received any previous treatment, 8 had undergone occlusion therapy only, 3 had received other forms of therapy, and 2 had previously received both occlusion therapy and other treatments. Classification of amblyopia subtypes and treatment history documentation followed the Clinical Guidelines for the Diagnosis and Treatment of Amblyopia in Children (China, 2021).

Furthermore, 2 experienced optometrists conducted comprehensive eye examinations for all participants as per some inclusion and exclusion criteria (Textbox 1).

**Table 1.** Demographic and baseline characteristics of participants.

Patient	Age (years)	Sex	logAE^a^	logFE^b^	Acc_AE^c^	Acc_FE^d^	Acc_BE^e^	Supp^f^	Stereo^g^
1	7	Male	0.30	0.10	2	1	1	OD^h^	800
2	9	Male	0.52	0.10	0	0	0	normal	1200
3	7	Male	0.52	0.10	0	0	0	normal	1200
4	7	Female	0.52	0.10	1	2	0	normal	1200
5	9	Male	0.30	0.00	7	6	7	normal	100
6	9	Female	0.30	0.10	0	0	0	normal	600
7	12	Female	0.52	0.10	0	0	0	OS^i^	1200
8	7	Female	1.00	0.10	0	0	0	OS	1200
9	8	Female	0.30	0.10	0	0	4	normal	1200
10	9	Female	0.70	0.10	0	0	0	normal	1200
11	7	Male	0.30	0.05	5	4	2	normal	200
12	8	Male	0.40	0.05	0	0	8	normal	200
13	12	Female	0.40	0.10	2	2	0	diplopia	1200
14	7	Male	0.30	0.05	0	0	0	normal	40
15	9	Female	0.30	0.05	0	0	0	normal	100
16	8	Male	0.30	0	4	2	3	normal	60
17	7	Female	0.30	0.05	3	2	0	normal	200
18	7	Female	0.30	0.10	1	3	2	normal	100
19	7	Female	0.30	0.05	7	8	7	normal	100
20	7	Female	0.40	0.05	2	0	1	normal	200
21	8	Female	0.40	0.10	2	6	3	OD	2400
22	7	Female	0.40	0.10	0	0	0	OD	1200
23	7	Female	0.40	0.10	0	0	0	normal	1200
24	7	Female	0.52	0.10	0	1	0	normal	1200
25	9	Male	0.52	0.10	0	0	0	normal	1200

a logAE: visual acuity of the amblyopic eye.

b logFE: visual acuity of the fellow eye.

c Acc_AE: accommodative sensitivity of the amblyopic eye.

d Acc_FE: accommodative sensitivity of the fellow eye.

e Acc_BE: binocular accommodative sensitivity.

f Supp: suppression.

g Stereo: stereopsis.

h OD: oculus dexter.

i OS: oculus sinister.

Textbox 1. **Inclusion and exclusion criteria.**

**Inclusion criteria**

- Aged between 7 and 12 years (this relatively broad age range was chosen to reflect real-world clinical referrals of late-childhood amblyopia; however, developmental heterogeneity across 7‐12 y was not powered for formal subgroup analysis and is considered in the interpretation of results).
- Best-corrected visual acuity in 1 eye, confirmed by cycloplegic refraction, was below 0.8, with an interocular visual acuity difference of ≥0.2 logMAR.
- Visual acuity remained stable without significant fluctuation over the past 3 months.
- No other ocular diseases or neurodevelopmental disorders were present.
- Consent to participate and provision of written informed consent.

**Exclusion criteria**

- Developmental delay or cognitive impairment that prevented compliance with experimental procedures.
- Presence of retinal disease or ocular media opacity (eg, cataract or corneal opacity).
- Uncorrectable vision problems.
- Ongoing atropine treatment during the study period or use of atropine within the past 3 months.
- Premature birth ≥8 weeks.
- Presence of severe concurrent ocular or systemic diseases.

### Game Principles and Design

This study was conducted in the visual function laboratory of the ophthalmology department at Luohu People’s Hospital, Shenzhen, Guangdong, China. Participants underwent visual function training at this location for a duration of 3 months.

While extensive research has been conducted on PL and stereoscopic vision training, few studies have integrated the design principles of these 2 approaches into a single game tailored for children.

In the design of serious games, the parallax effect generated by red-blue glasses (left red, right blue) is used for training. After wearing these special glasses, the left eye can only see the red image, and the right eye can only see the blue image. By controlling the size of the parallax, this method helps patients train and enhance their stereoscopic vision abilities. Using the physical light-splitting principle of the red-blue glasses, the left eye of the wearer receives only the red image, while the right eye sees only the blue image. The red and blue channels of each game element are extracted separately using Adobe Photoshop’s channel extraction function, ensuring that each frame displays 2 independent images. These 2 images are then horizontally displaced to create different levels of parallax, thereby achieving the effect of stereopsis training. Furthermore, to accommodate users with varying levels of stereopsis ability, the game includes a feature to adjust the size of the parallax: when a user has weaker stereoscopic vision (larger random-dot stereograms [RDS] value), a larger parallax is provided to reduce the difficulty, while a smaller parallax is used to increase the challenge for users with stronger ability (smaller RDS value).

The primary purpose of these 2 games is to effectively train children’s ability to discern objects at varying depth positions through carefully designed task mechanics that focus children’s attention on depth perception. These games were designed and developed by the research team using the Mugeda platform (Beijing Lexiang Yunchuang Technology Co, Ltd) and are specifically configured to run on the Magic Eye (Shenzhen Vizhi Holographic Technology Co, Ltd) hardware system. Both “Panda Eats Bamboo” and “Fishing Master” (developed by our research team) have been certified as class II medical devices by the National Medical Products Administration of China (Registration xiangxiezhuzhun20232210101).

**Figure 1.**
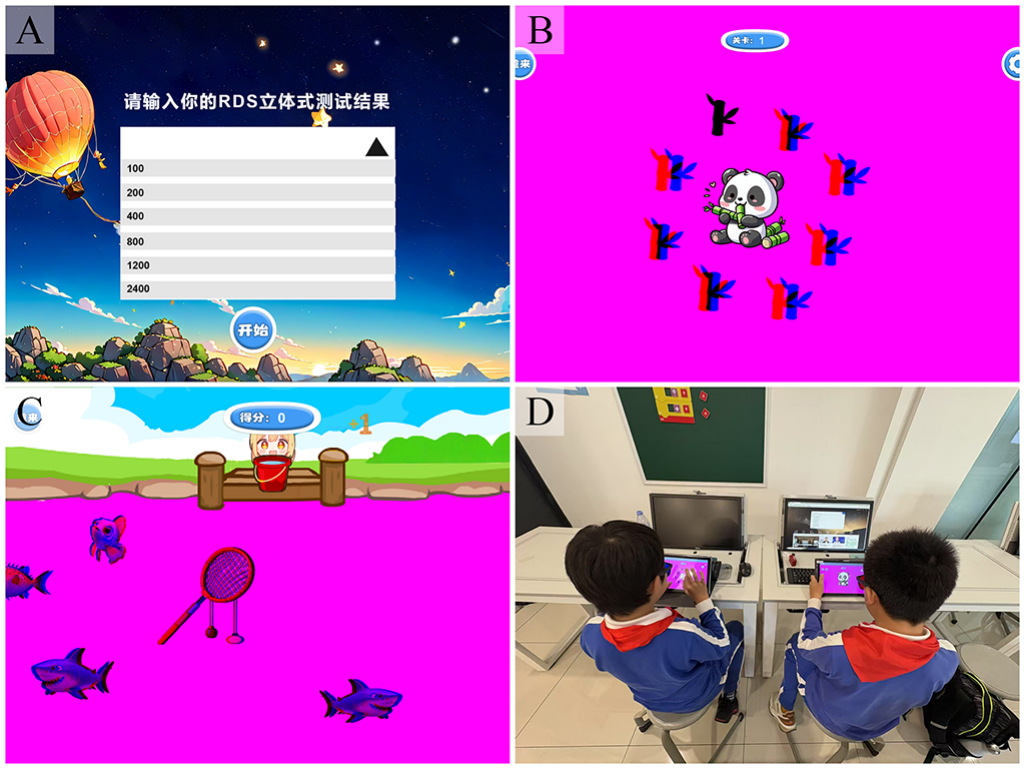
(A) Initial interface: selecting stereo level [屏幕中文： “选择立体等级”→英文“Select stereo level”]; (B) Panda Eats Bamboo [“开始”“下一关”→“Start”“next”]; (C) Fishing Master [“投网”“计分”→“cast”“Score”]; (D) Training with the Magic Eye device.

Game 1, “Panda Eats Bamboo” (Figure 1B), guides children’s attention explicitly toward evaluating depth differences among bamboo sticks, using their simple and repetitive circular motion as the training stimulus. The straightforward regularity and clear depth differences among the bamboo sticks make this game an ideal tool for depth perception training (refer to Multimedia Appendix 1 for the operational demonstration video). The design emphasizes helping children concentrate on depth judgments rather than complex gameplay mechanics or rules. Children aged 7‐12 years generally possess adequate visual capacity to engage with and benefit from this structured perceptual training.

Game 2, “Fishing Master” (Figure 1C), achieves this by requiring players to concentrate on judging depth perception. It trains children’s ability to distinguish objects at varying depths through a fishing mechanism. The dynamic characteristics of fish as moving targets enhance interactivity and engagement, effectively drawing children’s attention to depth cues (refer to Multimedia Appendix 2 for the demonstration video). Players must accurately perceive and react to the fish’s depth and location to successfully complete the catching action, which not only trains their spatial perception but also enhances their ability to discern changes in depth within dynamic scenarios.

The task design of this study was informed by the principles of PL and stereoscopic vision training, emphasizing adaptive task difficulty, enhancement of binocular disparity cues, and engagement with dynamic targets, with the goal of activating plastic changes in the visual cortex. For example, when children repeatedly judge the depth of objects, neurons in the primary visual cortex and secondary visual area (V1 and V2) regions gradually enhance their ability to integrate and respond to binocular disparity signals. This training prompts neurons in the V1 area to readjust their receptive fields, allowing for more efficient encoding of an object’s spatial position [39]. At the same time, dynamic tasks (such as tracking moving fish) activate the middle temporal visual area region in the brain’s dorsal pathway, which specializes in processing depth information of moving objects. Repeated training strengthens the sensitivity of neurons in this area to motion disparity, thereby improving the ability to predict the depth of fast-moving objects [40]. Additionally, decision-making tasks (such as selecting the raised bamboo) enhance the collaboration between the prefrontal cortex and the cerebellum, helping children integrate higher-level attention control into their visual judgments [41]. The brain’s white matter remodeling after training also supports functional improvement. For example, the corpus callosum fibers connecting the left and right visual areas become more efficient, promoting the integration of information from both eyes [42]. The task design mechanisms described above are based on theoretical frameworks proposed in previous literature; this study did not directly measure these neural pathways and therefore does not draw any causal or temporal conclusions regarding them.

### Training Device: Magic Eye

Magic Eye is a standard tablet computer provided by Shenzhen Vizhi Holographic Technology Co, Ltd. The device is equipped with an octa-core processor and a naked-eye 3D LCD display and supports TF card and USB storage accessories, WLAN connectivity, and 3.5 mm stereo headphone output. Unlike conventional tablets, it features specialized stereoscopic display technology that allows interaction with games on the tablet. For amblyopia treatment, patients need to wear red and blue anaglyph glasses to achieve the stereoscopic effect necessary for therapeutic intervention. The choice of this platform was based on its wide availability and common use in local hospitals and clinics, rather than any commercial relationship, and no financial or commercial ties exist between the investigators and the supplier. Magic Eye consists of 4 modules. The perception training module enables interaction with games, facilitates game switching, and allows for the adjustment of parallax sizes. The eye movement detection module monitors user gaze behavior, detecting whether the user is focused on the screen and providing prompts if the user’s attention deviates. The parameter control module provides unified management of user parameters with global accessibility, enabling reading and writing of fixed-format files and supporting remote updates or modifications of parameters by doctors or staff. The server management module logs detailed user training data, including training dates, activities, duration, and other relevant information.

### Experimental Procedure

**Figure 2.**
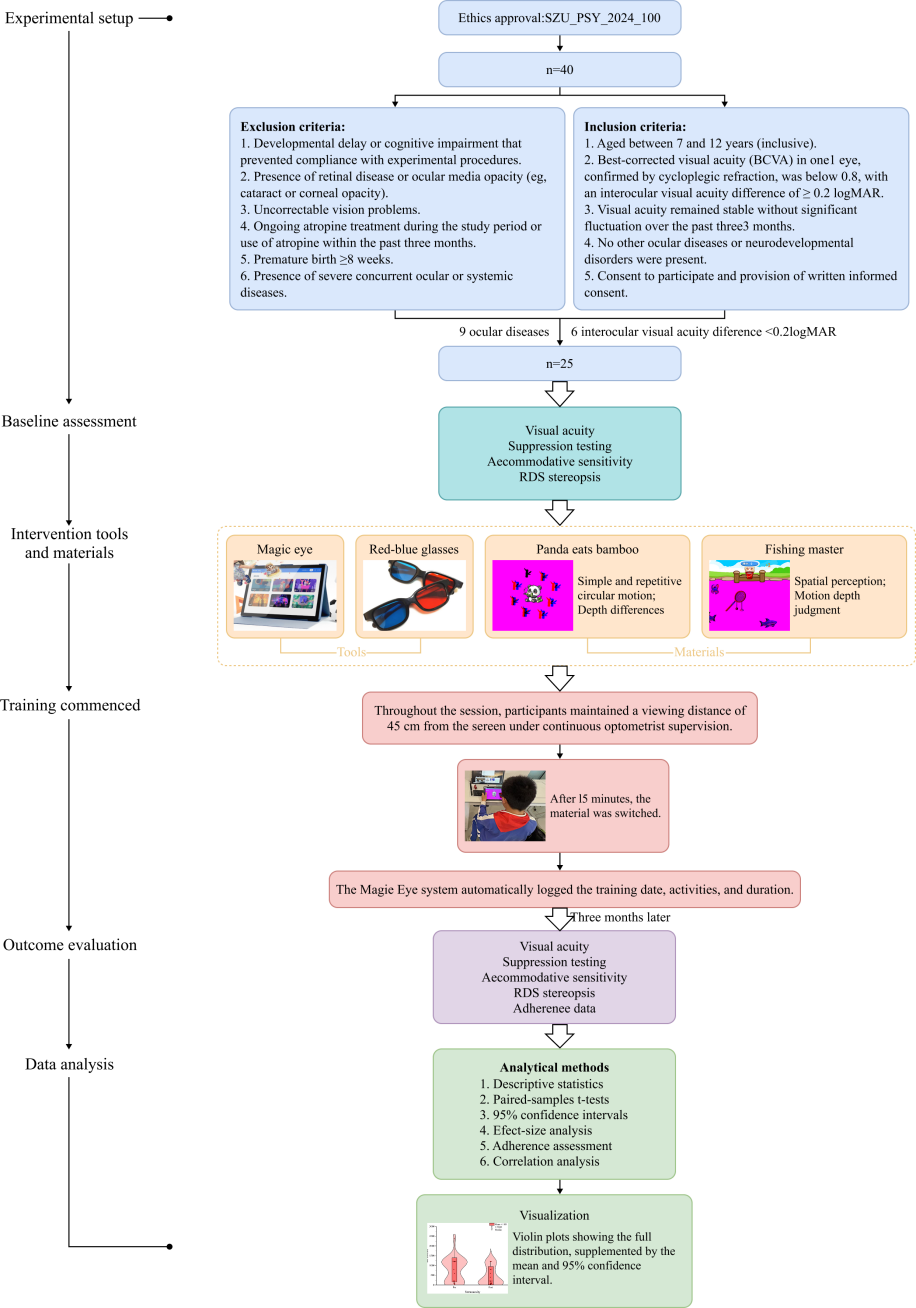
Experimental procedure. This flowchart illustrates the experimental design and workflow of the study on amblyopia treatment using serious games. Participants were screened according to inclusion and exclusion criteria, underwent baseline visual function assessments, and then participated in the Magic Eye training program, which included multiple game-based visual tasks. The system automatically logged training data, and posttraining evaluations were conducted to assess visual outcomes and adherence. RDS: random-dot stereograms.

Before the experiment, optometrists briefly introduced the use of Magic Eye to parents and participants. Visual training lasted 12 weeks, with 4 sessions per week, each session lasting 30 minutes. During each 30-minute session, the 2 games were played consecutively, with each game lasting 15 minutes. Training data, including training dates, activities, and duration, were collected through the Magic Eye system during each session. During training, participants were required to maintain a distance of approximately 45 cm from the device. All training was supervised by optometrists to ensure compliance with the protocol. A scheduled follow-up examination (including assessments of VA, accommodative function, stereopsis, and suppression) was arranged 6 months after the completion of training (Figure 2). This study reports only the immediate pre- and postintervention outcomes; follow-up data will be reported separately according to the preregistered protocol. To ensure that participants could independently complete the training, the serious games used in the experiment were briefly explained before the training sessions began. During each session, optometrists accompanied the participants, providing assistance and observing their performance when necessary.

### Outcome Measures

In binocular vision function assessment for children with amblyopia, various indicators play distinct roles in evaluating binocular coordination, accommodative ability, and stereoscopic vision.

#### Visual Function Data

VA was measured using the Tumbling E chart, which is suitable for children and patients unfamiliar with Roman letters. The Tumbling E chart consists of uppercase “E” shapes oriented in various directions [43]. Participants identified the orientation of the E as directed by the optometrist. VA measurements were converted to the LogMAR scale using the formula LogMAR=−log(VA) [44,45].

Suppression testing is a common clinical method for evaluating binocular vision function, primarily detecting monocular suppression or diplopia. The Worth 4 dot test is frequently used to assess simultaneous vision and fusion [46]. By identifying the number of dots seen, clinicians evaluate binocular vision function and disorders such as esotropia and exotropia [47].

Participants wore red-blue stereoscopic glasses and maintained a stable head position during the test. They first covered 1 eye and reported the number of dots seen, then uncovered the eye and reported the images perceived. Normal binocular vision participants see 4 dots; left-eye suppression patients see only 2 dots, indicating the left eye cannot coordinate with the right; right-eye suppression patients see 3 dots, suggesting a lack of coordination in the right eye. Patients with diplopia may see 5 dots, indicating poor coordination and possible esotropia or exotropia [47]. Suppression training, which involves repeated desuppression exercises, is critical for improving fusion ability and enhancing simultaneous and fusion vision [48].

Accommodative sensitivity testing assesses monocular and binocular accommodative function. Monocular sensitivity reflects the quality of accommodative function, while binocular sensitivity indicates the interaction between accommodation and convergence. Accommodative ability tests are used to evaluate this function [49]. This study followed the protocol outlined by García et al [50] to measure monocular and binocular accommodative ability.

Using ±2.00 D lens combinations, the speed at which participants could view targets (size 20/30) at 40 cm was measured in cycles per minute (CPM). The ±2 D flipper used in this study allowed adjustment of interpupillary distance from 44 mm to 89 mm and had a total weight of 40 g, making it lightweight and suitable for children to handle, thereby minimizing potential ergonomic effects on CPM scores. Participants reported the direction of targets circled in red. Correct responses resulted in lens flips, while incorrect responses were recorded as CPM within 1 minute. Nontested eyes were covered during monocular testing. The standard values for CPM are ≥11 for monocular testing and ≥8 for binocular testing. Values below these thresholds indicate accommodative insufficiency [51,52].

RDS stereoscopic vision tests assess binocular stereopsis, which represents a high level of binocular cooperation and is vital for children’s spatial cognitive development. RDS is commonly used to evaluate stereoscopic vision in both pediatric and adult ophthalmology [53].

This study used the Randot Preschool Stereotest to measure stereoscopic acuity, with the minimum disparity at which correct shapes could be identified serving as the indicator [27]. The normal range for stereoscopic acuity is 40‐60 arcseconds, with values exceeding 60 arcseconds considered abnormal [54].

#### Compliance Data

Adherence data were captured automatically via the Magic Eye platform. The system continuously logged session start and end times, effective training minutes (excluding pauses and background operation), training frequency, game type, level progression, and task difficulty; it also recorded interaction metrics (touch count, reaction time, and accuracy) and attention-quality indicators (gaze ratio and idle ratio). From these raw data, we derived the following adherence indices: completion rate (sessions completed÷prescribed sessions), duration rate (effective minutes÷prescribed total minutes), weekly adherence (proportion of weeks meeting the prescribed frequency), and quality-adjusted adherence (effective minutes weighted by attention-quality factors and normalized to percentage). All sessions were screened by predefined quality control rules: sessions with effective time <5 minutes or >10 minutes of continuous idle were labeled invalid and excluded.

### Statistical Analysis

For a 2-tailed paired design (α=.05, 1−*β*=0.80) with n=25, the minimum detectable within-participants effect is Cohen *d*_*z*≈0.58, a measure of the standardized effect size for paired samples (noncentral-*t*; normal approximation gives 0.56). Observed accommodative effects (Cohen *d*_*z*≈1.1‐1.4) exceed this threshold, whereas BCVA and stereoacuity show small-to-medium signals that warrant confirmation in controlled trials.

### Data Analysis

Data analysis was performed using IBM SPSS software. Descriptive statistics and paired *t*-tests were used to examine within-group differences, while CIs and effect sizes were calculated to quantify the magnitude and reliability of these differences. Results were visualized using violin plots. Furthermore, Pearson correlation analysis was conducted to explore the relationships between improvements in amblyopic eye VA and measures of accommodative function, stereopsis, and compliance, thereby revealing the statistical associations among these variables. Analyses were restricted to baseline and immediate postintervention outcomes; prespecified durability end points at 6 months were not analyzed in this manuscript.

## Results

### Overview

The preintervention and postintervention changes in the primary outcomes are summarized in Table 2, followed by detailed statistical analyses and graphical representations for each outcome in subsequent sections.

**Table 2.** Postintervention changes in primary outcomes (for accommodative sensitivity, negative Δ values reflect postintervention increases (improvement), as differences were computed as pre-post).

Parameter	Preintervention, mean (SD)	Postintervention, mean (SD)	Cohen *d* (95% CI)	*t* test (*df*)	*P* value
Log_AE^a^	0.42 (0.16)	0.37 (0.18)	0.96 (0.03 to 0.08)	4.82 (24)	<.001
Acc_AE^b^ (CPM^c^)	1.44 (2.18)	4.96 (2.91)	−1.36 (−4.59 to −2.45)	−6.81 (24)	<.001
Acc_FE^d^ (CPM)	1.48 (2.28)	3.76 (2.35)	−1.00 (−3.22 to −1.33)	−4.99 (24)	<.001
Acc_BE^e^ (CPM)	1.52 (2.49)	5.08 (2.71)	−1.11 (−4.88 to −2.24)	−5.56 (24)	<.001
Stereo^f^ (arc sec)	780.00 (613.57)	448.80 (472.23)	0.69 (134.24 to 528.16)	3.47 (24)	.002
Supp^g^ (n, % normal)	19 (76%)	24 (96%)	—^h^	—	—

a Log_AE: visual acuity of the amblyopic eye.

b Acc_AE: accommodative sensitivity of the amblyopic eye.

c CPM: cycles per minute.

d Acc_FE: accommodative sensitivity of the fellow eye.

e Acc_BE: binocular accommodative sensitivity.

f Stereo: stereopsis.

g Supp: suppression.

h Not applicable.

### Changes in VA

**Figure 3.**
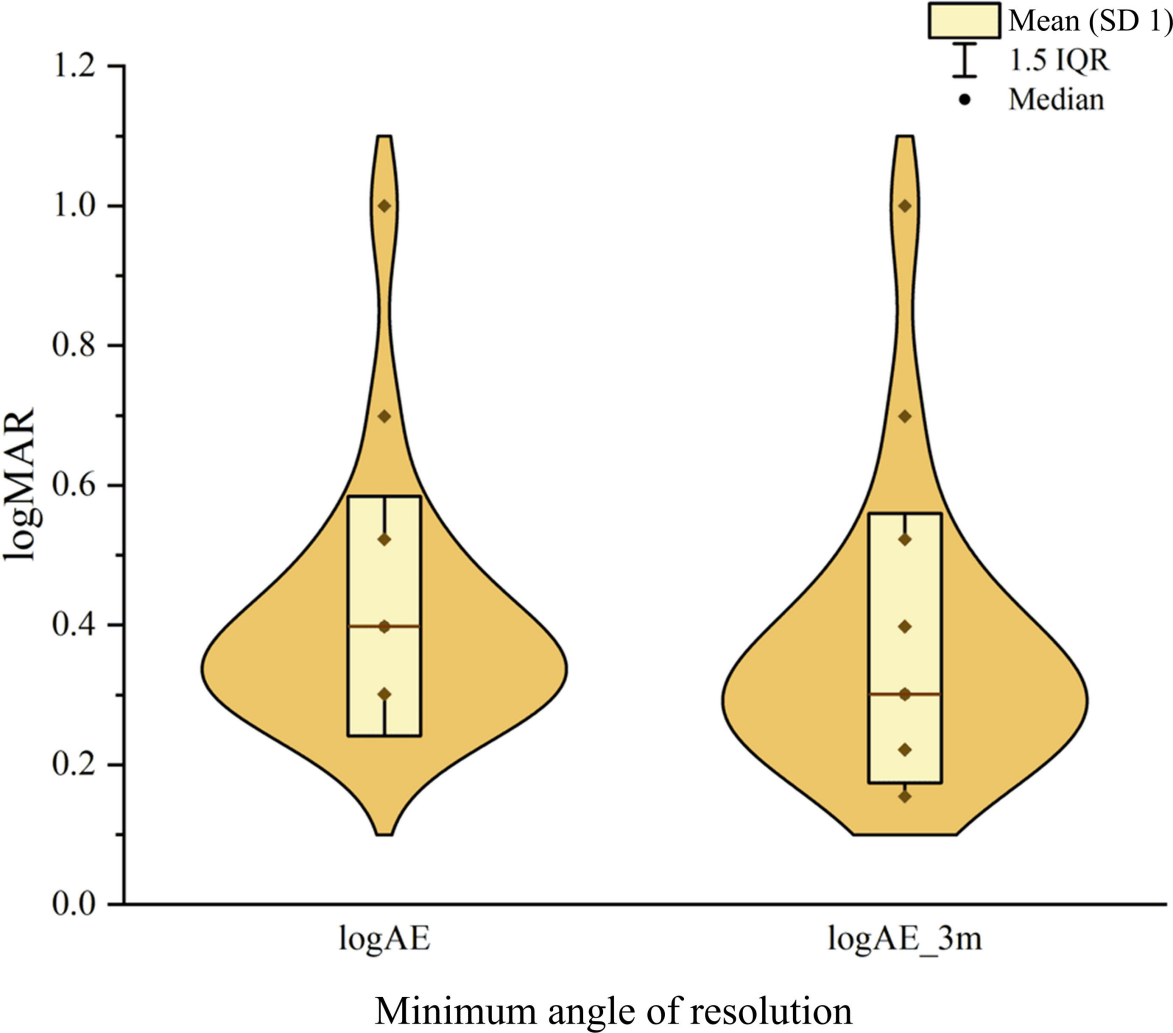
Improvement in visual acuity of children with amblyopia before and after treatment. logAE: visual acuity of the amblyopic eye.

Following the intervention, the amblyopic eye showed a significant improvement in logMAR VA (Figure 3). The mean VA of the amblyopic eye improved from 0.42 (SD 0.16) at baseline to 0.37 (SD 0.18) after the intervention, with a mean difference of 0.05. A paired-sample *t*-test revealed a *t*_24_ value of 4.82 (*P*<.001) and a 95% CI of 0.03‐0.08, indicating a highly significant difference. Effect size analysis yielded a Cohen *d* of 0.96, suggesting a large improvement with clear clinical relevance.

### Accommodative Sensitivity

**Figure 4.**
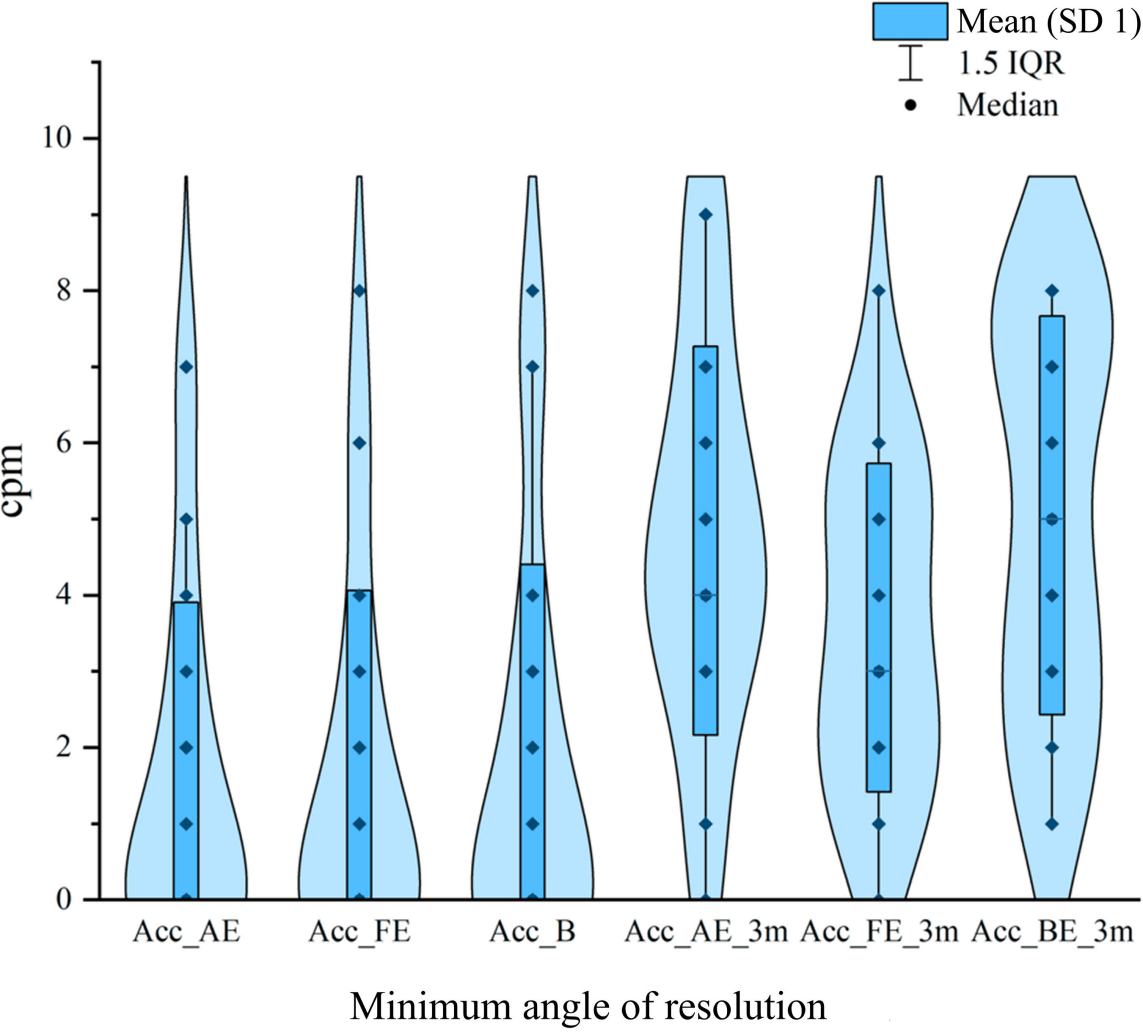
Improvement in binocular accommodative function of children with amblyopia before and after treatment. Acc_AE: accommodative sensitivity of the amblyopic eye; Acc_BE: binocular accommodative sensitivity; Acc_FE: accommodative sensitivity of the fellow eye; cpm: cycles per minute.

Following the intervention, both monocular and binocular accommodative abilities improved significantly (Figure 4). For the amblyopic eye, the mean accommodative sensitivity increased markedly from 1.44 (SD 2.18) before the intervention to 4.96 (SD 2.91) after the intervention, with a mean difference of –3.52. A paired *t*-test revealed a *t*_24_ value of –6.81 (*P*<.001) and a 95% CI of –4.59 to –2.45. The effect size (Cohen *d*=–1.36) indicated a large and clinically meaningful improvement in accommodative function of the amblyopic eye.

In the fellow eye, accommodative sensitivity also showed a significant enhancement, increasing from 1.48 (SD 2.28) to 3.76 (SD 2.35), with a mean difference of –2.28. The paired *t*-test yielded a *t*_24_ value of –4.99 (*P*<.001) and a 95% CI of –3.22 to –1.34, corresponding to a Cohen *d* of –1.00, suggesting a moderate-to-large facilitative effect on the fellow eye’s accommodation.

Furthermore, binocular accommodative sensitivity increased significantly from 1.52 (SD 2.49) to 5.08 (SD 2.71), with a mean difference of –3.56. The 95% CI ranged from –4.88 to –2.24, and the paired *t*-test yielded *t*_24_=–5.56 (*P*<.001). The effect size (Cohen *d*=–1.11) also reflected a substantial and clinically relevant improvement.

Overall, the intervention led to marked enhancements in both monocular and binocular accommodative abilities; improvement was greater in the amblyopic eye than in the fellow eye. These findings suggest that serious game–based visual training can effectively strengthen accommodative function in children and promote the recovery of binocular coordination.

### Stereoacuity

**Figure 5.**
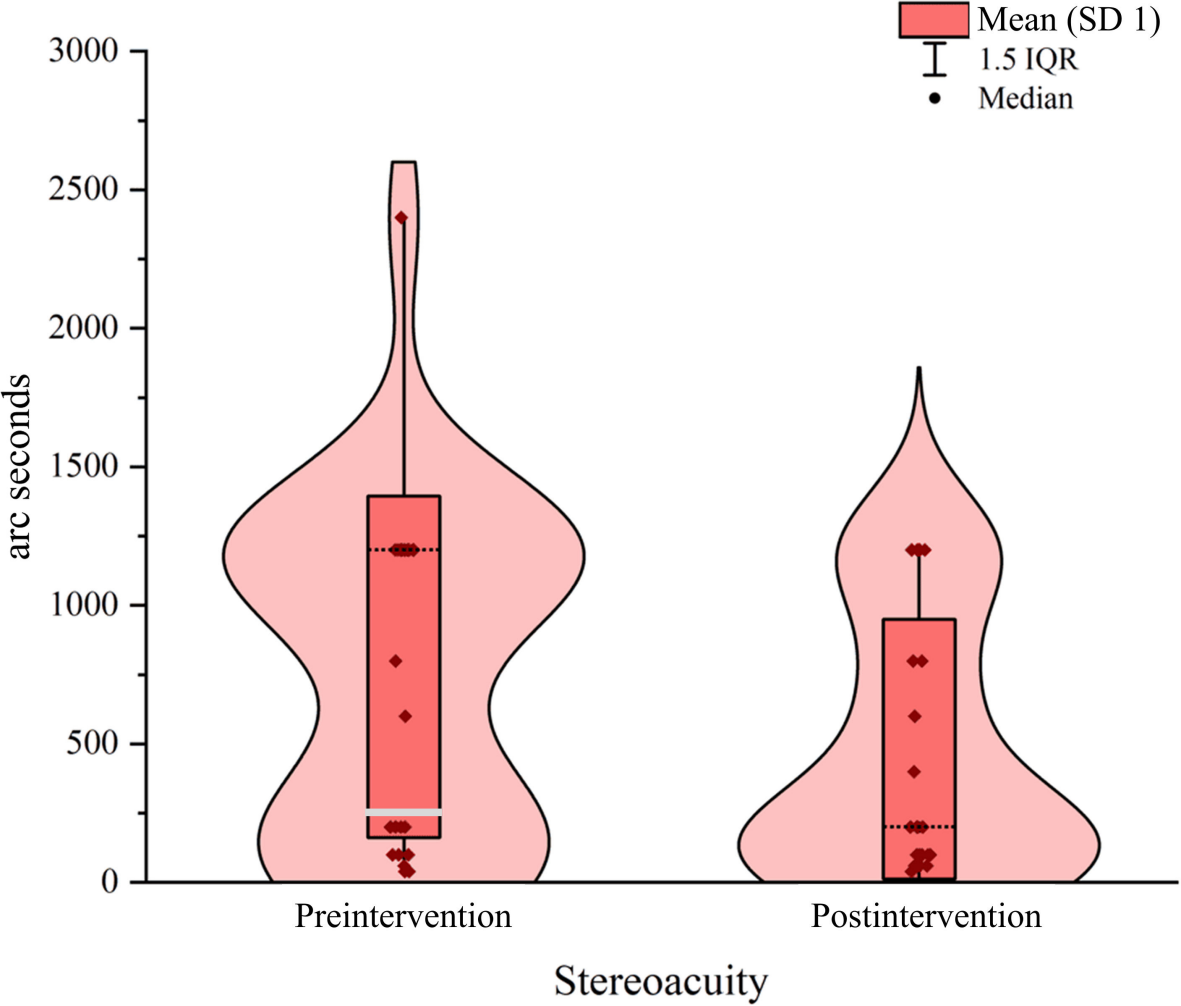
Improvement in stereopsis of children with amblyopia before and after treatment.

The intervention led to a significant improvement in stereoacuity, both statistically and clinically (Figure 5). Preintervention data indicated a mean stereoacuity of 780.00 (SD 613.57), which decreased significantly to 448.80 (SD 472.23) postintervention. This resulted in a mean difference of 331.20, with a 95% CI of 134.24-528.16. A paired *t*-test yielded a *t*_24_ value of 3.47 (*P*=.002), and Cohen *d* was 0.69, indicating a medium-to-large effect size. This demonstrates that the intervention had reliable statistical significance and certain clinical utility in improving participants’ stereoacuity.

Supporting these statistical findings, the violin plot illustrated a significant postintervention shift toward lower stereoacuity values, with the median and primary distribution range moving downward. Data points became more concentrated in the range representing better stereoacuity levels, reflecting consistent improvements in participants’ stereoacuity following the intervention.

### Suppression

Before the intervention, 19 of 25 participants (76%) showed normal Worth 4 dot responses. The remaining 6 participants (24%) had abnormal findings, including monocular suppression or diplopia. After the intervention, 24 of 25 participants (96%) demonstrated normal Worth 4 dot responses, with only 1 participant showing residual monocular suppression.

### Compliance Assessment

**Figure 6.**
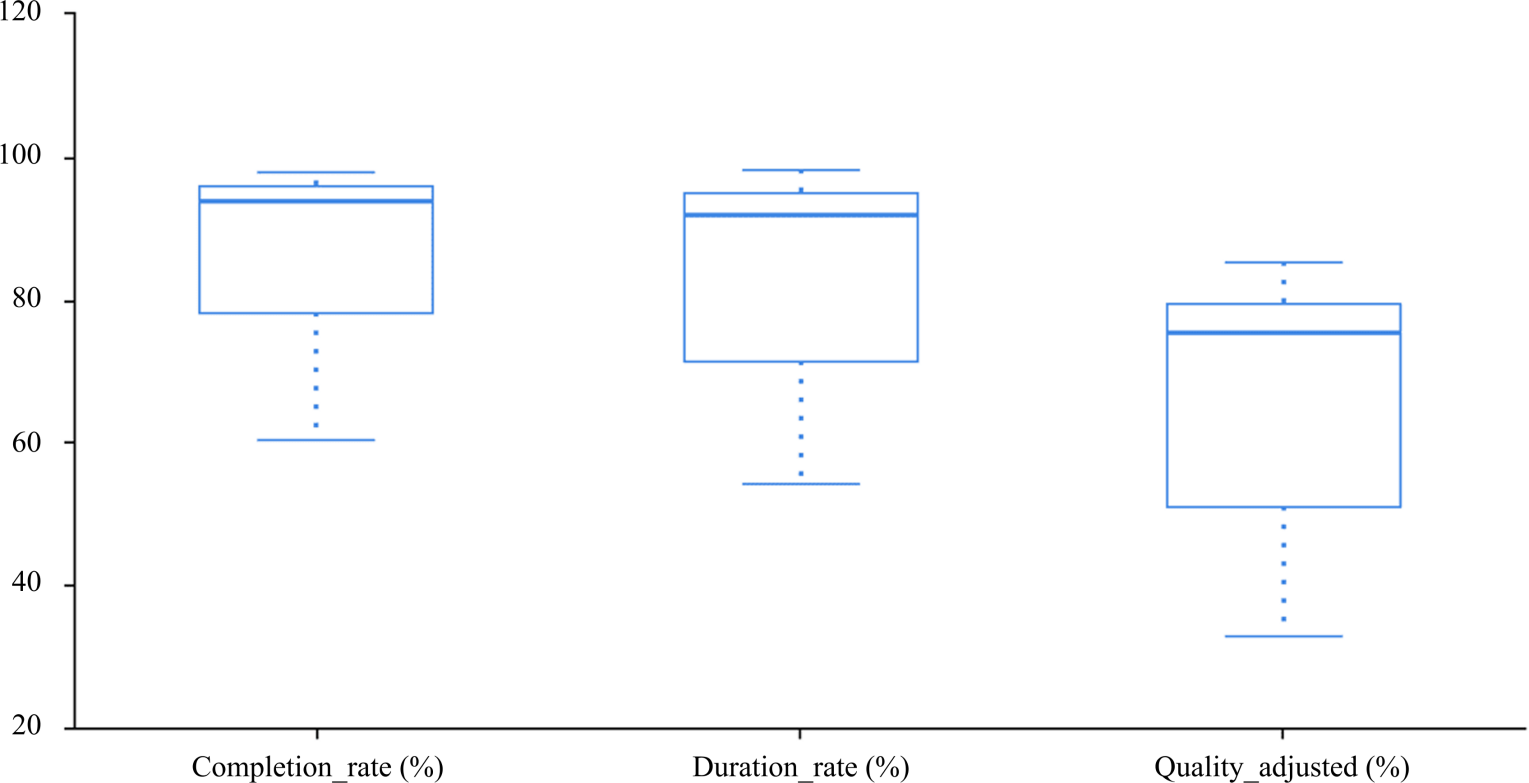
Overview of adherence metrics (completion, duration, and quality-adjusted).

A total of 25 participants completed the compliance and usage behavior assessments (Figure 6). Among the scheduled 48 training sessions, the mean number of completed sessions was 42.32 (SD 5.60). The cumulative effective training duration was 1229 (SD 201.07) minutes, out of a total planned duration of 1440 minutes. The mean weekly compliance rate was 83.04% (SD 12.68%). The mean fixation ratio was 0.85 (SD 0.10), and the mean inactivity ratio was 0.10 (SD 0.06). The three compliance indicators were as follows: (1) completion rate (mean 88.16%, SD 11.67%), (2) duration rate (mean 85.41%, SD 13.95%), and (3) quality adjusted (mean 68.57%, SD 16.4%).

### Correlation Analysis

**Figure 7.**
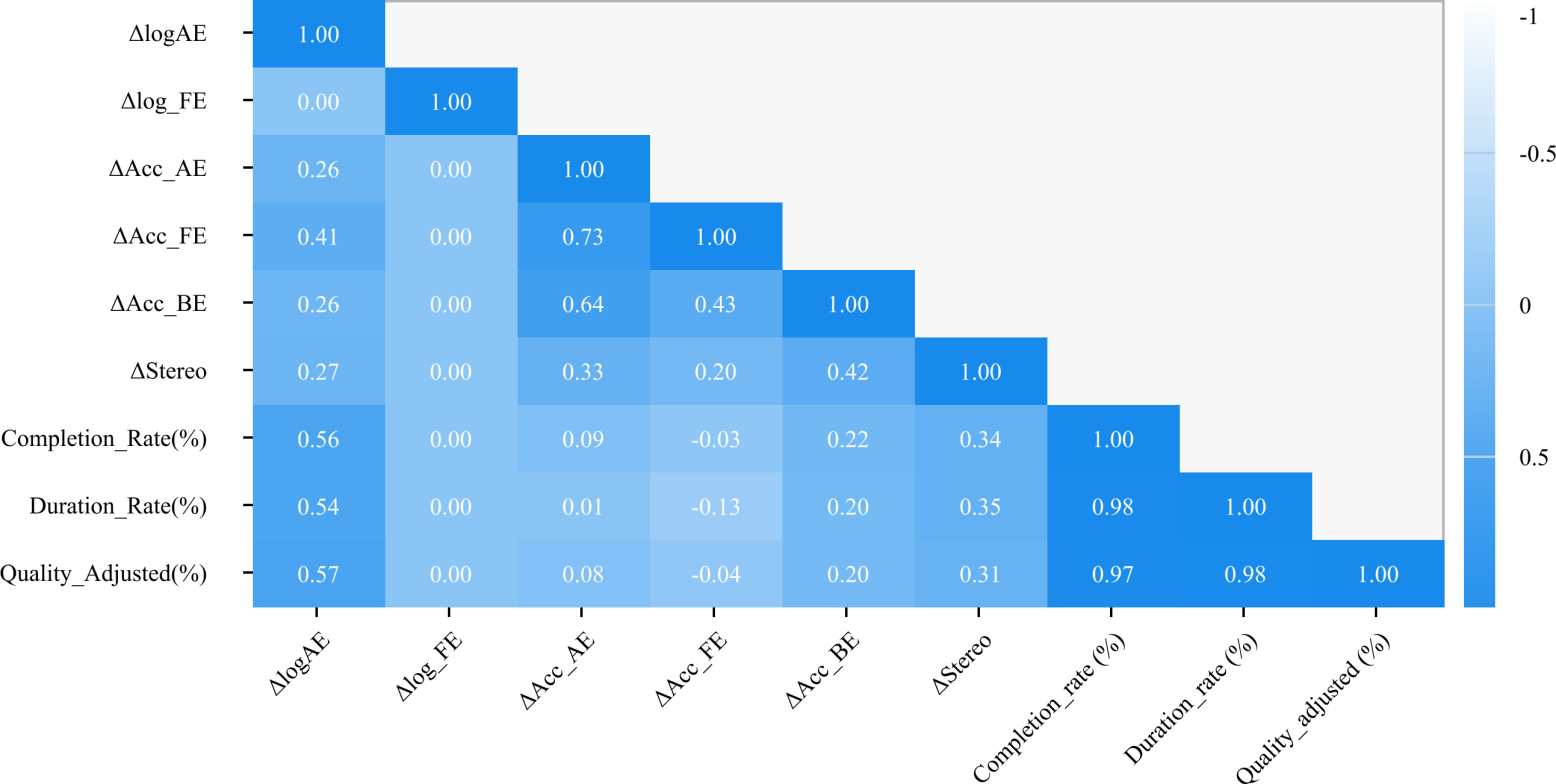
Pearson correlation heatmap. Acc_AE: accommodative sensitivity of the amblyopic eye; Acc_BE: binocular accommodative sensitivity; Acc_FE: accommodative sensitivity of the fellow eye; logAE: visual acuity of the amblyopic eye; logFE: visual acuity of the fellow eye.

Improvement in VA of the amblyopic eye was significantly positively correlated with the change in accommodative function of the fellow eye (*r*=0.41, *P*=.04), session completion rate (*r*=0.56, *P*=.004), effective training duration rate (*r*=0.54, *P*=.006), and quality-adjusted compliance (*r*=0.57, *P*=.003) (Figure 7). No significant correlations were observed between amblyopic eye visual improvement and changes in fellow-eye VA, amblyopic-eye accommodation, binocular accommodation, or stereopsis (all *P*>.05).

The compliance indicators were highly intercorrelated: session completion rate was strongly correlated with both effective training duration rate (*r*=0.98, *P*<.001) and quality-adjusted compliance (*r*=0.97, *P*<.001); similarly, effective training duration rate was highly correlated with quality-adjusted compliance (*r*=0.99, *P*<.001).

Regarding functional indices, changes in fellow-eye accommodation were positively correlated with both amblyopic-eye accommodation (*r*=0.73, *P*<.001) and binocular accommodation (*r*=0.43, *P*=.03). Additionally, changes in stereopsis were positively correlated with changes in binocular accommodation (*r*=0.42, *P*=.04). Given the number of pairwise correlations examined, these analyses are exploratory; no formal adjustment for multiple comparisons was applied, and marginal associations (particularly the correlation between BCVA improvement and fellow-eye accommodative sensitivity, *P*=.04) should be interpreted with caution.

## Discussion

### Principal Findings

In this single-arm feasibility study, 12-week serious-game training integrating PL and stereopsis tasks produced (1) a modest but statistically significant BCVA gain in the amblyopic eye (mean 0.05, SD 0.056 logMAR); (2) large within-participants improvements in accommodative sensitivity for the amblyopic, fellow, and binocular conditions (Cohen *d*_*z*≈1.36, 1.00, and 1.11, respectively); (3) a medium improvement in stereoacuity (Cohen *d*_*z*≈0.69); and (4) an increase in normal Worth 4 dot responses from 19/25 (76%) at baseline to 24/25 (96%) postintervention, with 5 of 6 participants with baseline suppression or diplopia showing normalization. Device-logged metrics indicated high adherence under supervised clinical conditions, and exploratory correlations suggested that greater adherence and changes in accommodative function were associated with VA gains. Given the single-group design, small sample, absence of multiple-comparison adjustment for the correlation matrix, and the heterogeneous 7‐12-year age range, these findings should be regarded as hypothesis-generating. The preliminary improvements observed in this late-childhood cohort are consistent with the presence of residual plasticity but require confirmation in age-stratified RCTs before firm conclusions can be drawn.

### Comparison With Previous Work

This study systematically evaluated the efficacy of gamified interventions in improving visual functions among children with amblyopia, validating the clinical applicability of integrating PL and stereopsis training strategies. The findings demonstrated that, compared with traditional treatments, the gamified intervention not only effectively enhanced basic VA but also improved accommodative function and showed notable efficacy in the restoration of stereopsis.

Compared with baseline, BCVA in the amblyopic eye improved from 0.42 (SD 0.16) to 0.37 (SD 0.18) logMAR (mean change=0.05 logMAR, *t*_24_=4.82, *P*<.001), corresponding to an improvement of approximately 0.5 lines on a logMAR chart. Because this study used a single-arm, pre-post feasibility design with clinic-based, optometrist-supervised sessions, the observed within-participants improvement should be interpreted cautiously. Nonspecific factors, such as practice effects on visual testing, natural maturation in children, and the Hawthorne effect associated with supervised participation, may have contributed to the change. Therefore, direct comparisons with RCTs are not appropriate; randomized, controlled studies with adequate sample sizes are required to establish comparative efficacy.

It is noteworthy that To et al [55] achieved a VA improvement of 0.17 logMAR in adult patients, which closely aligns with the results of this study. However, their research implemented a high-intensity training protocol (1‐2 h daily), which might be less feasible for children. In contrast, this study used shorter sessions through gamified design, limiting training to 30 minutes per session to accommodate children’s attention spans and ensure higher adherence. This approach not only enhanced children’s engagement but also maintained treatment effectiveness, suggesting that shorter, frequent sessions may be preferable for pediatric populations.

Moreover, the accommodation function, an important yet often overlooked factor in visual rehabilitation for amblyopia, significantly improved in response to gamified interventions, as indicated by the ±2.00 D flipper test results. The mean accommodative sensitivity increased markedly, with 6 of 25 participants (24%) achieving normal levels (>8 CPM), representing a substantial improvement compared with baseline. Accommodative function is likely due to the dynamic focusing tasks embedded within the game mechanics, requiring rapid identification of variable virtual elements, thus effectively training the responsiveness of the ciliary muscles. Unlike previous studies, such as those by Herbison et al [56] and To et al [55], which did not specifically quantify accommodative sensitivity due to their focus on dichoptic stimuli alone, this study fills this critical gap and highlights the necessity of including dynamic focusing mechanisms in amblyopia training programs.

Regarding stereopsis, the median stereoacuity improved significantly from 780.00 arc seconds to 448.80 arc seconds (*t*_24_=3.47, *P*=.002), and the RDS test pass rate increased from 2 of 25 participants (8%) to 6 (24%). Such consistent improvements were not observed in previous studies; for example, the Frisby stereoacuity test used by Herbison et al [56] showed no statistically significant enhancement, likely due to the majority of their participants having strabismic amblyopia without targeted fusion training. Similarly, To et al [55] reported improved stereopsis using polarized dichoptic Tetris, but their improvements did not surpass the achievements observed in this study’s targeted, dynamic, and repetitive training approach.

Despite promising outcomes, this study’s generalizability is limited by its single-center, small-sample design (n=25). Future research should incorporate advanced technologies, such as VR, and adopt multicenter RCT designs to further validate long-term efficacy. Additionally, exploring advanced display technologies, like VR, and conducting multicenter RCTs could further verify the long-term effectiveness of this approach. Future research might also quantify neuroplasticity maintenance thresholds (eg, monthly BCVA decay <0.02 logMAR) through multicenter longitudinal studies and establish personalized retraining recommendation systems based on artificial intelligence prediction models. To accommodate cognitive differences across age groups, developing age-specific game content libraries could be beneficial. Moreover, the simplicity of the current games introduces a potential learning curve effect, where initial performance gains may partly reflect increased familiarity with the tasks rather than true visual improvement. In addition, outcome measures were collected only at baseline and posttraining, without interim assessments. This limits the ability to characterize the trajectory of visual improvement or disentangle transient learning effects from long-term treatment gains. Future studies should incorporate intermediate testing sessions to better capture the temporal dynamics of therapeutic response. Ultimately, these efforts aim to transition amblyopia management toward a comprehensive “technology-physiology-behavior” multidimensional intervention paradigm.

This study demonstrated that improvements in amblyopic eye VA were moderately and positively correlated with compliance-related indicators, including session completion rate, effective training duration rate, and quality-adjusted compliance—all of which reached statistical significance. This finding suggests that compliance is a key driver of short-term visual gains. The result is consistent with previous multicenter studies emphasizing that the efficacy of home-based binocular training is often constrained by adherence. However, large RCTs have not observed a clear dose–response relationship in actual data—training duration, or the magnitude of interocular contrast adjustment was not significantly associated with VA improvement [11]. These results indicate that good compliance is a necessary condition for benefit, but a “more-is-better” linear dose effect has not been substantiated.

In contrast, no significant correlation was found between improvement in amblyopic eye VA and changes in stereopsis. This finding aligns with several pediatric studies reporting that improvements in VA and stereopsis during short-term binocular training may occur asynchronously. Considering the correlation pattern observed in this study (nonsignificant association between VA and stereopsis changes), our results are consistent with previous findings. However, because this study did not include multiple timepoint analyses, the temporal sequence of these effects could not be determined, and the underlying mechanisms require further validation through prospective longitudinal data. Notably, stereopsis improvement correlated only with changes in binocular accommodation, not with VA improvement, suggesting that binocular coordination mechanisms—such as binocular accommodation and fusion—may play a more direct role in stereopsis recovery, rather than being mediated by monocular VA enhancement.

A moderate correlation was identified between improvement in BCVA and changes in fellow-eye accommodative sensitivity (*r*=0.41, *P*=.04), whereas no significant association was found with amblyopic-eye accommodative changes (*P*>.05). This pattern appears counterintuitive, as improvements in amblyopic-eye VA would typically be expected to relate to changes within the same eye. One possible interpretation is that binocular coordination or interocular facilitation mechanisms might manifest initially through compensatory adjustments in the fellow eye. However, given the small and clinically heterogeneous sample, this correlation may also represent a statistical artifact or reflect measurement variability. Accordingly, the result should be interpreted with caution, and future studies with larger samples and objective physiological assessments are needed to clarify the underlying mechanisms.

Overall, the correlation patterns observed in this study support the following interpretation: in home-based gamified binocular training, compliance serves as the critical threshold determining whether measurable improvement occurs, yet evidence for a linear dose–response effect remains insufficient. The restoration of stereopsis appears to rely more on binocular coordination and suppression-rebalancing mechanisms than on simple monocular VA enhancement. Unlike large-scale RCTs that reported no overall superiority or dose–response effect, our findings reveal individual-level positive associations detectable by statistical modeling—highlighting a practical direction for optimizing therapeutic efficacy through improved engagement and training quality.

All training sessions in this feasibility study were conducted under optometrist supervision in the clinic to ensure compliance and protocol fidelity. Therefore, the high adherence observed likely reflects the benefits of supervised conditions and may not directly translate to unsupervised home environments. Although the Magic Eye platform is designed to support remote logging and could potentially enable home-based deployment in the future, this feasibility phase did not evaluate such functionality. Future studies should explicitly test unsupervised, home-based implementation to determine whether similar adherence and efficacy can be maintained outside the clinic.

The current framework provides preliminary evidence that a gamified binocular training system can be feasibly implemented in clinical settings. With further validation and appropriate safety and monitoring mechanisms, this approach could be extended to establish a scalable “home training–community screening–hospital evaluation” network, potentially reducing clinical workload and improving accessibility in resource-limited regions. However, claims regarding reduced hospital visits or real-world cost-effectiveness remain hypothetical and require confirmation in prospective trials incorporating economic evaluation and unsupervised home-based use.

### Limitations

This study has 3 primary limitations. First, as a single-center, single-arm, pre-post feasibility study with a small and clinically heterogeneous sample (n=25), this work cannot exclude practice or learning effects as alternative explanations for the observed improvements, thereby limiting causal inference. The limited sample size also reduced statistical power and precluded subgroup analyses, which may have obscured potential differences in treatment responsiveness across amblyopia subtypes and treatment histories. To mitigate bias, standard refractive correction was maintained throughout, and analyses were restricted to within-group changes and correlations without making superiority claims over patching or atropine. Future research will use multicenter randomized or stratified controlled trials with appropriate comparator arms (patching, atropine, nongamified, or sham conditions) and independent data monitoring to strengthen causal inference. Second, outcome assessments were limited to baseline and the 12-week end point, which precludes characterization of trajectories and durability and prevents establishing the temporal sequence of short-term learning versus longer-term retention; a 6-month follow-up has been prespecified, and subsequent studies will add interim assessments at weeks 2, 4, and 8 and extend follow-up to ≥6‐12 months. Third, the intervention bundles multiple components (binocular rebalancing, disparity-based training, and gamification for motivation), making it difficult to isolate the specific active ingredients; in this report, gamification is conservatively treated as an adherence-enhancing strategy rather than an independent therapeutic mechanism. Future studies will use factorial or multiarm designs (eg, gamified vs nongamified interactive, stereoscopic vs nonstereoscopic, and monocular vs binocular) to disentangle mechanisms and test both independent and interactive effects. In addition, the inclusion of children aged 7‐12 years introduces developmental heterogeneity in residual visual plasticity and treatment responsiveness. The sample size in this study was underpowered for age-stratified analyses; consequently, the reported effects should be interpreted as averaged signals across a late-childhood cohort rather than uniform efficacy at each age, and future trials should incorporate prespecified age strata.

### Conclusions

This study integrated PL with stereoscopic perception training within a serious game intervention administered to children aged 7‐12 years with amblyopia over 12 weeks (4 sessions per wk, 30 min each). Within a clinically feasible time frame, it achieved significant improvements in multidimensional visual functions: VA in the amblyopic eye showed modest yet statistically significant improvement, accommodation agility markedly increased, stereopsis demonstrated moderate optimization, and overall suppression status improved, with normal Worth 4 dot responses increasing from 19/25 to 24/25. Platform-based logging demonstrated good adherence and effective training duration under fully supervised in-clinic conditions, indicating that the protocol is feasible and monitorable in a controlled clinical setting. Whether similar adherence can be maintained in unsupervised home environments remains unknown and should be explicitly evaluated in future studies. Potential benefits related to affordability and resource efficiency remain hypothetical and require confirmation through dedicated economic evaluations and comparative studies. Collectively, serious game-based training offers a promising complementary therapeutic pathway for pediatric amblyopia. Objective compliance data also provides an evidence base for individualized prescription refinement and future multicenter RCTs, informing dose setting and methodological optimization.

## Supplementary material

10.2196/77402 Multimedia Appendix 1 Video of “Panda Eats Bamboo” operational demonstration.

10.2196/77402 Multimedia Appendix 2 Video of “Fishing Master” operational demonstration.

## Abbreviations

BCVA: best-corrected visual acuity
CPM: cycles per minute
PL: perceptual learning
RCT: randomized controlled trial
RDS: random-dot stereograms
VA: visual acuity
VR: virtual reality
